# Clinical, biochemical and molecular characterization of Korean patients with mucolipidosis II/III and successful prenatal diagnosis

**DOI:** 10.1186/s13023-016-0556-2

**Published:** 2017-01-17

**Authors:** Mina Yang, Sung Yun Cho, Hyung-Doo Park, Rihwa Choi, Young-Eun Kim, Jinsup Kim, Soo-Youn Lee, Chang-Seok Ki, Jong-Won Kim, Young Bae Sohn, Junghan Song, Dong-Kyu Jin

**Affiliations:** 1 Department of Laboratory Medicine and Genetics, Samsung Medical Center, Sungkyunkwan University School of Medicine, 81 Irwon-ro, Gangnam-gu, Seoul 06351 Republic of Korea; 2 Department of Pediatrics, Samsung Medical Center, Sungkyunkwan University School of Medicine, 81 Irwon-ro, Gangnam-gu, Seoul 06351 Republic of Korea; 3 Department of Medical Genetics, Ajou University Hospital, Ajou University School of Medicine, Suwon, Republic of Korea; 4 Department of Laboratory Medicine, Seoul National University College of Medicine, Seoul National University Bundang Hospital, Seongnam, Republic of Korea

**Keywords:** *GNPTAB*, Lysosomal storage disease, Mucolipidosis, Prenatal diagnosis

## Abstract

**Background:**

Mucolipidosis types II and III (ML II/III) are autosomal recessive disorders caused by a deficiency in the lysosomal enzyme N-acetylglucosamine-1-phosphotransferase. We investigated the molecular genetic characteristics of the *GNPTAB* gene, which codes for the alpha/beta subunits of a phosphotransferase, in Korean ML II/III patients. We included prenatal tests and evaluated the spectrum of mutations in East Asian populations with ML II/III through a literature review.

**Methods:**

Seven patients from six families were enrolled in the study including two prenatal tests using chorionic villi samples. A diagnosis of ML II/III was made based on clinical findings and increases in serum lysosomal enzyme levels. PCR and direct sequencing were performed to identify *GNPTAB* mutations.

**Results:**

We found 14 mutant alleles including seven known mutations of c.2189delT (p.Leu730fs*7), c.1090C > T (p.Arg364*), c.2681G > A (p.Trp894*), c.3565C > T (p.Arg1189*), c.310C > T (p.Gln104*), c.1071G > A (p.Trp357*) and c.2574_2575delGA (p.Asn859Glnfs*2). Four were novel variants of unknown significance: c.992A > G (p.Tyr331Cys), c.2666 T > A (p.Leu889*), c.637-6 T > G (p.Thr213Phefs*11), and c.471_472delTT (p.Tyr158Serfs*8). Family studies revealed the probands to be compound heterozygotes. The fetuses carried the same *GNPTAB* mutations as the mucolipidosis II/III probands in the prenatal diagnosis.

**Conclusions:**

We identified *GNPTAB* mutations in all patients with ML II/III, but did not identify a hot spot in Korean patients. We successfully performed prenatal diagnosis using molecular investigation.

## Background

Mucolipidosis type II/III (ML II/III) is an autosomal recessive inborn error resulted from the accumulation of lysosomal substrates. This rare metabolic disorder is caused by a deficiency of UDP-GlcNAc-1-phosphotransferase, an enzyme responsible for the synthesis of mannose-6-phosphate (M6P), a recognition marker in the cis part of the Golgi apparatus [1, 2]. The loss of M6P recognition results in impaired trafficking of lysosomal hydrolases and eventual lysosomal dysfunction.

ML was initially described in 1967 as a disease similar to mucopolysaccharidosis (MPS) that presented with different features such as low urinary acid mucopolysaccharides and abundant inclusions in fibroblasts [3]. For this reason, ML II wasoriginally called Inclusion Cell or I-Cell disease. ML II is characterized by coarse facial features, short stature, hyperplastic gums, organomegaly, and retarded psychomotor development [4]. ML III, originallyknown as pseudo-Hurler polydystrophy, was described Hurler-like disorder without mucopolysacchariduia [5]. ML III is a milder disorder with attenuated characteristics and survival to adult life. It is allelic to ML II and has a closely related pathogenesis. GlcNAc-1-phosphotransferase is absent in MLII and lacking in ML III. Intermediate forms of ML II and III have been previously described [6]. GlcNAc-1-phosphotransferase is composed of alpha, beta, and gamma subunits, and ML II/III is caused by mutations in *GNPTAB,* which encodes for the alpha and beta subunits of the phosphotransferase [7].

The diagnosis of ML II/III is based on clinical, radiological, biochemical, and molecular findings. Of these, a mutation study of *GNPTAB* is important in terms of confirmation of disease and prediction of prognosis. In addition, a molecular analysis of *GNPTAB* is essential for prenatal diagnosis of ML II/III [8]. In the present study, we investigated *GNPTAB* mutations in five patients with ML II/III and successfully performed prenatal testing in two pregnant women. In addition, genotypes in Korean patients with ML II/III and the *GNPTAB* mutation spectrum in East Asian populations with ML II/III were studied through a literature review.

## Methods

### Patients

Seven individuals from six families were enrolled in the study, including two obtained via prenatal genetic testing using chorionic villi samples (CVSs). Prenatal genetic test was done in case 3 and 4 because they had affected older siblings. In addition, familial studies in cases 3, 5, 6 and 7 were performed. The median age of probands at diagnosis was three years (range, 0.5–7 years), and only one patient was male. A diagnosis of ML II/III was performed based on clinical symptoms and abnormal findings of lysosomal enzyme levels in blood. The study was approved by the Institutional Ethics Committee of Samsung Medical Center (IRB #2015-11-120-001).

### Enzyme assays

Lysosomal activity, including that of β-hexosaminidase, β-glucosidase, α-N-acetylglucosaminidase, in the plasma and leukocytes was measured using methylumbelliferyl substrates in spectrofluorometry (Beckman Coulter DU-650, Fullerton, CA) at wavelengths of 360 and 448 nm. Arysulfatase A was measured using the substrate p-nitrocatechol sulfate. Enzyme activities of Cases 5 and 6 were measured at an outside hospital.

### PCR and sequencing

Genomic DNA was extracted from peripheral blood leukocytes with the Wizard Genomic DNA Purification Kit (Promega, Madison, WI, USA) according to the manufacturer’s recommendations. All 21 exons along with flanking regions of the *GNPTAB* gene were amplified using PCR with primers (Table 1) designed by the authors (Model 9700: Applied Biosystems, Foster City, CA, USA). For reverse transcription PCR (RT-PCR), cDNA corresponding to exons 6–8 was amplified with primers (forward: actaaggatgttgaagatgccc, reverse: tcctgcttagactggctgatg) by use of cDNA Synthesis Kit (Applied Biosystems). The amplified products were sequenced using the BigDye Terminator Cycle Sequencing Ready Reaction Kit (Applied Biosystems) on an ABI Prism 3100 Genetic Analyzer (Applied Biosystems). Nucleotides were numbered according to the corresponding GenBank accession number of *GNPTAB* (NM_024312.4).

**Table 1:** Sequences of Primers Used for PCR amplification and Sequencing of GNPTAB

Exon	Primers
1	F: ctatgcccctccgtcctc
	R: gctcaggagttcgagaccag
2	F: ttgtccttttcaggaactgtagc
	R: cacaggggccacactaatct
3	F: cccccagctacagtttgaa
	R: acctccacctcccaaagttc
4	F: ggccaccttatattggagca
	R: actctaaccctccccagtgc
5	F: tccatgagataaaagtcttcatttg
	R: gcagctgttttgcttctcttt
6	F: tcccatgaagaattcccttt
	R: gcatcacaacacaagcttcaa
7	F: gctgtttttctttgagaatcttttt
	R: aaggagtgaggctcttctgg
8	F: ggaggttgaggtgagcagag
	R: taccaaaccaatggcagtga
9	F: aatgctgtctctttgaattttgg
	R: gagagctgtttgggtttggt
10	F: ccctttacccttctacctcca
	R: tatgcttcccaagctggtct
11	F: tcaacgcagcaggatctaaa
	R: actcctcccagctcagcttt
12	F: tgatccagcctcctctgc
	R: cctcttcagtgatttatgttgttctc
13_1	F: cacaaggacgacatgcaaat
	R: cgtaacccttctgggctgta
13_2	F: tgatccttctcccaaaccag
	R: tgatctcagcaaggctgact
13_3	F: aggcggaaatcctttttgag
	R: aatcagagatgggggctttt
13_4	F: gctccacaggaaaaacaggt
	R: aaatgaaaccatgtaagaaaagca
14	F: tgacccgttaacatgtatttca
	R: catttgcagagatggacttttt
15	F: tgctcgtgtttgagttgtttg
	R: ggttggtctcgaactcctga
16	F: ttggcattgtctcattctgc
	R: ttacgcatctatggggtgaa
17	F: ggtttggtttgtgaaaaatgc
	R: ccgtagtggactcaacatcca
18	F: aatcacaaaggtctggcttttt
	R: atgggggaccctatctcaac
19	F: tcattcccccagagaatcat
	R: aggttgcagtgagctgaggt
20	F: cctctctcctgcctggataa
	R: tgctgcctgaatattgtgaaa
21	F: ttttggaagaggaatgatgga
	R: aggatgacaggtccatgagc

Novel missense variation mutations were analyzed for mutational possibility using *in silico* analysis through Polymorphism Phenotyping-2 (PolyPhen-2) [9] and Sorting Intolerant from Tolerant (SIFT) [10]. The 1000 Genomes Project data [11], the Exome Aggregation Consortium [12], and the NCBI database of Single Nucleotide Polymorphisms (dbSNPs) were checked for known sequence variants. Evolutionary conservation of amino acid residues in various species was assessed using the EVOLA website (http://www.h-invitational.jp/evola_main/annotation.cgi?hit=HIT000331461).

## Results

The common disease-related symptoms at diagnosis were short stature, multiple joint contracture, coarse face, and developmental delay. Patients with ML II showed typical coarse face: flat face, shallow orbits, depressed nasal bridge, prominent mouth, and gingival hypertrophy. Patients with ML III showed mild coarse face without gingival hypertrophy. Only Patient 7 showed hepatosplenomegaly. Patient 1, 2, 5, 6, and 7 showed mild thickness of heart valves. Patients with ML II showed remarkably height less than −2.2 standard deviation score (SDS), and patients with ML III showed relatively short stature (−2 ~ −0.5 SDS). Clinical information and identified mutations are summarized in Table 2.

**Table 2:** Clinical characteristics and *GNPTAB* mutations in seven patients with ML II/III

Case no.	Phenotype	Age at Dx. (month)	Sex	Symptoms at diagnosis	Nucleotide change Nucleotide change	Amino acid change
1	III	3.10	M	growth retardation, joint contracture, developmental delay	c.992A > G c.2189delT	p.Tyr331Cys p.Leu730fs*7
2	II	0.5	F	developmental delay, synostosis, puffy face	c.1090C > T c.2666 T > A	p.Arg364* p.Leu889*
3^a^	NA	NA	F	normal, prior affected sibling	c.2681G > A c.3565C > T	p.Trp894* p.Arg1189*
4^a^	NA	NA	F	normal, prior affected sibling	c.310C > T c.3565C > T	p.Gln104* p.Arg1189*
5	III	7.3	F	mental retardation, asymmetric chest, joint contracture,	c.637-6 T > G c.2574_2575delGA	p.Thr213Phefs*11 p.Asn859Glnfs*2
6	III	6.3	F	joint contracture	c.637-6 T > G c.2574_2575delGA	p.Thr213Phefs*11 p.Asn859Glnfs*2
7	II	1.8	F	growth retardation, joint contracture, puffy face, hepatosplenomegaly	c.471_472delTT c.1071G > A	p.Tyr158Serfs*8 p.Trp357*

^a^Prenatal test, *Dx* diagnosis, *NA* not applicable

**Fig. 1:**
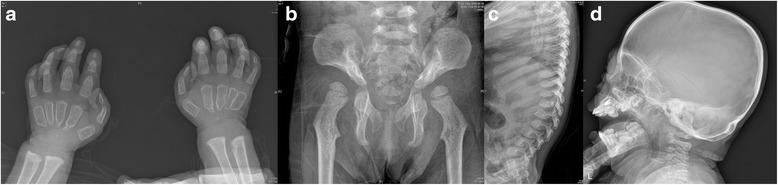
Radiographs of 21-month-old Korean girl with ML II. **a** Radiograph of hands and wrists showing broad and under-modelled proximal phalanges (*bullet-shaped*) and proximal pointing of metacarpals. **b** Radiograph of pelvis and proximal femurs showing dysplasia/resorption of the lower third of the ilia femoral heads and femoral necks and e. Clierd’s crook deformity.” **c** Radiograph of spine showing thoracolumbar kyphosis and beaking of the vertebrae. **d** Radiograph of skull showing J-shaped sella turcica

**Fig. 2:**
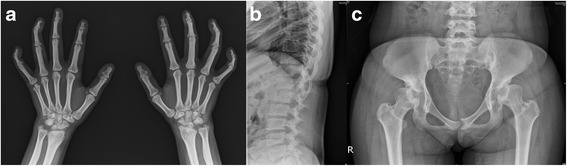
Radiographs of 19-year-old Korean female with ML III. **a** Radiograph of hands and wrists showing claw hands: impossible flexion and maximum extension without objective skin thickening or joint involvement. Small and irregular carpal bones and wide proximal phalanges are noted. **b** Radiograph of spine showing irregularity of the vertebral end plates. **c** Radiograph of pelvis showing flattened acetabulum and femoral head deformity

The representative radiographs of ML II and III are shown in Figs. 1 and 2, respectively. Radiographs of patient 7 with ML II at the age of 21 months show features reminiscent of “osteitis fibrosa cystica” in Fig. 1. Erosive changes, especially in the hands and hips, are seen (Fig. 1a, b). The proximal phalanges are broad and under-modelled. The proximal metacarpals show a mixture of features of osteodystrophy and dysostosis multiplex, becoming eroded and narrowed to a point. The carpal bones are osteopenic and hypoplastic. There is also over-modelling of the long bones and bowing of the proximal end of the femur leading to coxa valga or a or s also over-modelling of the long bones and bowing of the proximal hypoplastic and resorbed (Fig. 1b). In addition, there is osteopenia of the spine. The spine shows thoracolumbar kyphosis, beaking of the vertebrae (Fig. 1c), and the skull shows J-shaped sella turcica (Fig. 1d), which are typical of lysosomal storage disorders with skeletal involvement.

Radiographs of patient 6 with ML III at the age of 19 years are shown in Fig. 2. The characteristic radiologic findings of the hands are small and irregular carpal bones and relatively wide proximal phalanges (Fig. 2a). In the lumbar spine, irregular delineation of the vertebral bodies is seen (Fig. 2b). In the pelvis, progressive hip dysplasia with a flattened acetabulum and femoral head destruction are seen (Fig. 2c). The plasma levels of lysosomal enzyme activities were measured, excluding those in Cases 3 and 4, and are listed in Table 3. The plasma activity of lysosomal hydrolases tested showed a 5- to 25-fold increase in upper reference limit as follows: Arysulfatase A (x25), α-N-acetylglucosaminidase (x5 ~ 26), and β-hexosaminidase (x7 ~ 24). Otherwise, the activities of none of these enzymes were increased in leukocytes.

**Table 3:** Lysosomal enzyme activities in patients with ML II/III

	Case no.					Reference range	Reference range ^a^
	1	2	5^a^	6^a^	7	(nmol/hr/mg)	(nmol/min/mL)
Arylsulfatase A							
Plasma	847	312	42	43	2228	NA	0.1–1.6
Leukocyte	66	26	NA	NA	38	25–80	NA
α-N-Acetylglucosaminidase							
Plasma	234	NA	9	NA	NA	22.3–60.9	0.1–0.6
Leukocyte	0.93	NA	NA	NA	NA	0.90–1.51	NA
β-Hexosaminidase							
Plasma	4520	12247	14	13	8196	374–666	0.5–3.1
Leukocyte	728	482	NA	NA	358	611–991	NA
β-Glucosidase							
Plasma	0.7	0.4	NA	NA	0.86	NA	NA
Leukocyte	7.0	9.2	NA	NA	8.3	5.1–11.32	NA
β-Glucuronidase							
Plasma	NA	NA	18	19	NA	NA	0.1–2.0
Leukocyte	NA	NA	NA	NA	NA	NA	NA

*NA* not available; ^a^performed at outside hospital

**Fig. 3:**
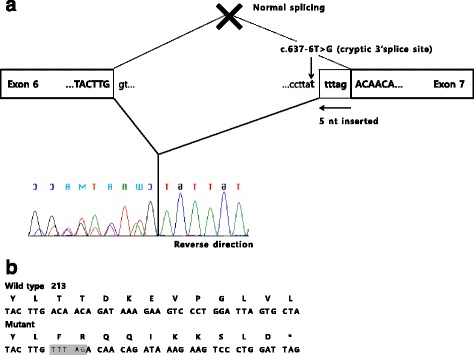
Molecular effects of the c.637-6 T > G mutation. **a** A schematic representation and cDNA sequence from case 6. **b** The predicted amino acid sequence (inserted nucleotides are shaded)

Fourteen mutant alleles including seven known (c.2189delT (p.Leu730fs*7), c.1090C > T (p.Arg364*), c.2681G > A (p.Trp894*), c.3565C > T (p.Arg1189*), c.310C > T (p.Gln104*), c.1071G > A (p.Trp357*) and c.2574_2575delGA (p.Asn859Glnfs*2)) and four novel (missense, nonsense, frameshift, and splicing mutations) mutations were detected. The four novel mutations were c.992A > G (p.Tyr331Cys), c.2666 T > A (p.Leu889*), c.637-6 T > G, and c.471_472delTT (p.Tyr158Serfs*8). All were absent in the healthy controls of the 1000 Genomes Project and Exome Aggregation Consortium data. Cases 5 and 6 are siblings; they are the second and third of three children, where the eldest child has a normal phenotype. The family study of Cases 5 and 6 revealed the mutation c.637-6 T > G in a father and the eldest sister, and c.2574_2575delGA in a mother. In order to determine a possible splicing aberration of c.637-6 T > G, RT-PCR sequencing in case 6 was performed. The result revealed that a new splice site was observed which leads to an insertion of 5 nucleotides and a premature stop codon (p.Thr213Phefs*11) (Fig. 3). A family study of Case 3 and 7 was also performed, which revealed the probands to be compound heterozygous. No family members who participated in the family study were affected individuals, and all had a heterozygous mutant allele. Cases 3 and 4 were prenatal tests with chorionic villi sampling in which the fetus carried the same *GNPTAB* mutations as the ML II/III probands.

All reported *GNPTAB* mutations in Korean patients with ML II/III are listed in Table 4. A total of 14 mutations have been reported in the Korean population, including those from this study. The most common type of mutation was a nonsense mutation (50%), followed by frameshift (31%), splicing (13%), and missense (6%) mutations. Frameshift mutation is the most common mutation in Asian populations (52%). All mutations reported in Asian populations are summarized in Table 5. The c.3565C > T (p.Arg1189*) mutation was most frequently observed in Korean and Japanese populations (11.5 and 32.5%).

**Table 4:** *GNPTAB* mutations in 13 Korean patients with ML II/III

Mutation type	Exon no.	Nucleotide change	Amino acid change	No. of alleles	Reference
Missense	9	c.992A > G	p.Tyr331Cys	1	This study
Nonsense	3	c.310C > T	p.Gln104*	2	[13], This study
Nonsense	9	c.1071G > A	p.Trp357*	1	This study
Nonsense	9	c.1090C > T	p.Arg364*	1	This study
Nonsense	13	c.2666 T > A	p.Leu889*	1	This study
Nonsense	13	c.2681G > A	p.Trp894*	2	[13], This study
Nonsense	15	c.3091C > T	p.Arg1031*	1	[14]
Nonsense	16	c.3173C > G	p.Ser1058*	1	[13]
Nonsense	19	c.3565C > T	p.Arg1189*	5	[13], This study
Frameshift	5	C.471_472delTT	p.Tyr158Serfs*8	1	This study
Frameshift	13	c.2189delT	p.Leu730fs*7	1	This study
Frameshift	13	c.2574_2575delGA	p.Asn859Glnfs*2	3	[13], This study
Frameshift	19	c.3456_3459dupCAAC	p.Ile1154Glnfs*3	1	[14]
Frameshift	19	c.3474_3475delTA	p.His1158fs*15	1	[13]
Splicing	IVS6	c.637-6 T > G	p.Thr213Phefs*11	2	This study
Splicing	IVS13	c.2715 + 1G > A	-	2	[13]

**Table 5:** Summary of all reported *GNPTAB* mutations in East Asian patients with ML II/III

Mutation type	Nucleotide change	Amino acid change	Exon no.	Reference	Allele frequency	Ethnicity
Missense	c.992A > G	p.Tyr331Cys	9	This study	0.9% (1/120)	KOR
Missense	c.1001G > T	p.Arg334Leu	9	[15]	0.9% (1/120)	JPN
Missense	c.1120 T > C	p.Phe374Leu	10	[15]	6.7% (8/120)	JPN
Missense	c.2866C > T	p.His956Tyr	14	[15]	1.8% (2/120)	JPN
Missense	c.3458A > G	p.Asn1153Ser	19	[15]	0.9% (1/120)	JPN
Nonsense	c.310C > T	p.Gln104*	3	[13, 15], This study	5.3% (6/120)	KOR, JPN
Nonsense	c.1071G > A	p.Trp357*	9	[16], This study	2.7% (3/120)	KOR, CHN
Nonsense	c.1090C > T	p.Arg364*	9	[16, 23], This study	5.9% (7/120)	KOR, CHN
Nonsense	c.2666 T > A	p.Leu889*	13	This study	0.9% (1/120)	KOR
Nonsense	c.2681G > A	p.Trp894*	13	[13, 15], This study	3.4% (4/120)	KOR, JPN
Nonsense	c.3091C > T	p.Arg1031*	15	[14]	0.9% (1/120)	KOR
Nonsense	c.3173C > G	p.Ser1058*	16	[13]	0.9% (1/120)	KOR
Nonsense	c.3565C > T	p.Arg1189*	19	[13, 15, 24], This study	32.5% (39/120)	KOR, JPN, CHN
Frameshift	c.471_472delTT	p.Tyr158Serfs*8	5	This study	0.9% (1/120)	KOR
Frameshift	c.914_915insA	p.Asp305fs	8	[15]	0.9% (1/120)	JPN
Frameshift	c.2089_2090insC	p.Leu697fs	13	[15]	1.8% (2/120)	JPN
Frameshift	c.2189delT	p.Leu730fs*7	13	This study	0.9% (1/120)	KOR
Frameshift	c.2422delC	p.Leu808fs*19	13	[24]	0.9% (1/120)	CHN
Frameshift	c.2427delC	p.Leu810fs	13	[15]	0.9% (1/120)	JPN
Frameshift	c.2544delA	p.Lys848fs	13	[15]	1.8% (4/120)	JPN
Frameshift	c.2574_2575delGA	p.Asn859Glnfs*2	13	[13], This study	2.5% (3/120)	KOR
Frameshift	c.2693delA	p.Lys898fs	13	[15]	0.9% (1/120)	JPN
Frameshift	c.3310delG	p.Ala1104fs	17	[15]	0.9% (1/120)	JPN
Frameshift	c.3388_3389insC + c.3390C > T	p.Val1130fs	18	[15]	0.9% (1/120)	JPN
Frameshift	c.3428_3429insA	p.Asn1143fs	18	[15]	0.9% (1/120)	JPN
Frameshift	c.3456_3459dupCAAC	p.Ile1154Glnfs*3	19	[14]	0.9% (1/120)	KOR
Frameshift	c.3474_3475delTA	p.His1158fs*15	19	[13]	0.9% (1/120)	KOR
Frameshift	c.3741_3744delAGAA	p.Glu1248fs	21	[15]	0.9% (1/120)	JPN
Frameshift	duplication exon2	Frameshift	2	[15]	5.0% (6/120)	JPN
Splicing	c.637-6 T > G	p.Thr213Phefs*11	IVS6	This study	1.8% (2/120)	KOR
Splicing	c.2715 + 1G > A	-	IVS13	[13, 15, 23]	7.5% (9/120)	KOR, JPN, CHN

*CHN* Chinese, *JPN* Japanese, *KOR* Korean

## Discussion

**Fig. 4:**
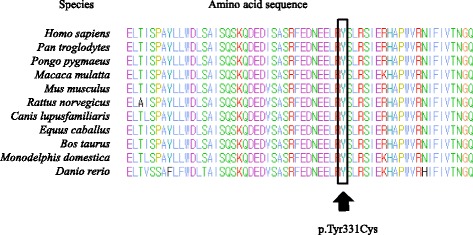
Evolutionary conservation of the amino acid residues of a novel missense, likely pathogenic variant. The affected residue, Tyr331, is strictly conserved in various mammals and zebrafish

In this study, five patients with ML II/III were confirmed through molecular genetic testing, and two cases were successfully screened using prenatal tests. A total of 14 mutant alleles were found in seven cases of ML II/III, and four of them were novel. Among four novel *GNPTAB* mutations, c.992A > G (p.Tyr331Cys) was the first missense variation detected in a Korean patient with ML II/III. There were no other missense variations in Korean ML II/III patients. The novel c.992A > G variation was considered to be a likely pathogenic variant because the effect of the amino acid change of p.Tyr331Cys was predicted to be “likely damaging” by PolyPhen-2 and “not tolerated” by SIFT. In addition, p.Tyr331 is a highly conserved region among several species (Fig. 4). Evolutionary conservation of the amino acid residues for p.Tyr331 has been observed in various mammals and zebrafish (*Homo sapiens, Pan troglodytes, Pongo pygmaeus, Macaca mulatta, Mus musculus, Rattus norvegicus, Canis lupusfamiliaris, Equus caballus, Bos Taurus, Monodelphis domestica,* and *Danio rerio*).

Another novel splicing mutation (c.637-6 T > G) was observed in two sisters (Cases 5 and 6). Their father and eldest sister were heterozygous carriers of the c.637-6 T > G mutation. RT-PCR sequencing revealed that this mutation created a new cryptic 3′ splice site resulting in truncated proteins. Although there have been some reports describing molecular genetic analyses of MLs, there were a few reports in the Korean population. No homozygotes were observed in the present study or in other studies on the Korean population [13, 14], while homozygotes have been detected in the Chinese and Japanese populations [15, 16].

To date, three cases in the Korean population have been identified to be compound heterozygotes for two nonsense mutations; both were patients with ML II. The affected children of the prenatal diagnosis cases (Cases 3 and 4), the probands reported by Paik et al. [13], had cardiac anomalies of coronary artery atresia or mitral valve prolapse and expired at 13 years and 3 years of age, respectively. In addition, compound heterozygotes for a nonsense and frameshift mutation also exhibited the ML II phenotype. On the other hand, patients that were heterozygous for at least one allele with a missense or splicing mutation demonstrated an attenuated phenotype. This genotype-phenotype correlation in ML II/III is compatible with the results described in previous reports [13, 17–19]. Genotypes with compound heterozygotes consisting of nonsense and frameshift mutations are expected to produce no RNA product, and they are associated with a more severe phenotype (ML II). Thus, phenotype seems to be well correlated with genotype in 13 Korean patients.

**Fig. 5:**
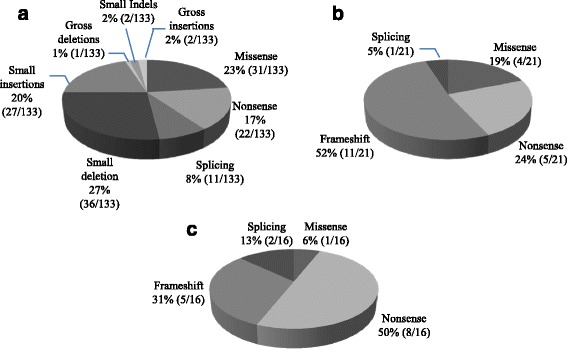
Summary of the reported *GNPTAB* mutation spectrum: **a** *GNPTAB* mutation types among all reported mutations in all ethnic populations, **b** *GNPTAB* mutation types identified in East Asian ML II/III patients other than Koreans, **c** *GNPTAB* mutation types identified in Korean ML II/III patients

According to the Human Gene Mutation Database, more than 130 *GNPTAB* mutations have been reported in patients with ML II/III [20]. Figure 5 shows the *GNPTAB* mutation spectrum according to ethnic population with ML II/III. A missense mutation is the most abundant type of mutation (27%), and a small deletion is the next most common (25%). In the Korean population, however, only one missense mutation (6%) has been observed to date, while nonsense mutations make up 50% of mutations [13, 14]. In a Japanese population, frameshifts were the most common mutation type (61%), and missense mutations were relatively common (22%); the frequently observed mutation c.3565C > T (p.Arg1189*) had an allele frequency of 32.5% [15]. In a Korean population, the most frequently observed mutation was c.3565C > T (p.Arg1189*) (11.5%, 5/26 alleles), excluding the overlapping case of siblings, but the difference in frequency compared to the second most common mutation (c.2574_2575delGA, 7.7%) was not large. The c.3503delTC mutation, known as a single causal mutation in a French-Canadian founder population [21], was the most frequently encountered mutation in the largest study in a Western population [19]. Considering that c.3503delTC has not been observed in Asian populations, the spectrum of mutation type seems to exhibit ethnic differences.

In this study, two prenatal tests with CVS were successfully performed, revealing the same mutations as those present in the probands. To the best of our knowledge, only one case has been published on the prenatal diagnosis of ML II/III by means of molecular study [8]. In Korea, a prenatal test of ML II/III is legal, and this is the first case of prenatal investigation of ML II/III. As there are currently no effective therapies such as hematopoietic stem cell transplantation [22], a prenatal test for this mutation is expected to simply provide information to the parents of probands with ML II/III.

## Conclusions

The direct measurement of UDP-GlcNAc-1-phosphotransferase is complicated and not available in many countries. In addition, enzyme assays might have some limitations in terms of interpretation. Thus, molecular genetic investigation is clinically very useful in the diagnosis of ML II/III patients. In this respect, *GNPTAB* genotyping is essential to confirm the diagnosis of ML II/III and is helpful for distinguishing carriers via prenatal diagnosis.

## Abbreviations

CVSs: Chorionic villi samples
M6P: Mannose-6-phosphate
ML II/III: Mucolipidosis type II/III
MPS: Mucopolysaccharidosis

## References

[1] Pohlmann R, Waheed A, Hasilik A, von Figura K (1982). Synthesis of phosphorylated recognition marker in lysosomal enzymes is located in the cis part of Golgi apparatus. J Biol Chem.

[2] Reitman ML, Varki A, Kornfeld S (1981). Fibroblasts from patients with I-cell disease and pseudo-Hurler polydystrophy are deficient in uridine 5′-diphosphate-N-acetylglucosamine: glycoprotein N-acetylglucosaminylphosphotransferase activity. J Clin Invest.

[3] Leroy JG, Demars RI (1967). Mutant enzymatic and cytological phenotypes in cultured human fibroblasts. Science.

[4] Okada S, Owada M, Sakiyama T, Yutaka T, Ogawa M (1985). I-cell disease: clinical studies of 21 Japanese cases. Clin Genet.

[5] Kelly TE, Thomas GH, Taylor HA, McKusick VA, Sly WS, Glaser JH (1975). Mucolipidosis III (pseudo-Hurler polydystrophy): Clinical and laboratory studies in a series of 12 patients. Johns Hopkins Med J.

[6] Poenaru L, Castelnau L, Tome F, Boue J, Maroteaux P (1988). A variant of mucolipidosis. II. Clinical, biochemical and pathological investigations. Eur J Pediatr.

[7] Kudo M, Brem MS, Canfield WM (2006). Mucolipidosis II (I-cell disease) and mucolipidosis IIIA (classical pseudo-hurler polydystrophy) are caused by mutations in the GlcNAc-phosphotransferase alpha/beta -subunits precursor gene. Am J Hum Genet.

[8] Alegra T, Koppe T, Acosta A, Sarno M, Burin M, Kessler RG (2014). Pitfalls in the prenatal diagnosis of mucolipidosis II alpha/beta: A case report. Meta Gene.

[9] PolyPhen: prediction of functional effect of human nsSNPs. http://genetics.bwh.harvard.edu/pph/. Accessed 1 May 2015.

[10] SIFT. http://sift.jcvi.org/. Accessed 1 May 2015.

[11] 1000 Genomes. A Deep Catalog of Human Genetic Variation. http://browser.1000genomes.org. Accessed 1 May 2015.

[12] ExAC Browser (Beta). Exome Aggregation Consortium. http://exac.broadinstitute.org/. Accessed 1 May 2015.

[13] Paik KH, Song SM, Ki CS, Yu HW, Kim JS, Min KH (2005). Identification of mutations in the GNPTA (MGC4170) gene coding for GlcNAc-phosphotransferase alpha/beta subunits in Korean patients with mucolipidosis type II or type IIIA. Hum Mutat.

[14] Heo JS, Choi KY, Sohn SH, Kim C, Kim YJ, Shin SH (2012). A case of mucolipidosis II presenting with prenatal skeletal dysplasia and severe secondary hyperparathyroidism at birth. Korean J Pediatr.

[15] Otomo T, Muramatsu T, Yorifuji T, Okuyama T, Nakabayashi H, Fukao T (2009). Mucolipidosis II and III alpha/beta: mutation analysis of 40 Japanese patients showed genotype-phenotype correlation. J Hum Genet.

[16] Yang Y, Wu J, Liu H, Chen X, Wang Y, Zhao M (2013). Two homozygous nonsense mutations of GNPTAB gene in two Chinese families with mucolipidosis II alpha/beta using targeted next-generation sequencing. Genomics.

[17] Tiede S, Storch S, Lubke T, Henrissat B, Bargal R, Raas-Rothschild A (2005). Mucolipidosis II is caused by mutations in GNPTA encoding the alpha/beta GlcNAc-1-phosphotransferase. Nat Med.

[18] Bargal R, Zeigler M, Abu-Libdeh B, Zuri V, Mandel H, Ben Neriah Z (2006). When Mucolipidosis III meets Mucolipidosis II: GNPTA gene mutations in 24 patients. Mol Genet Metab.

[19] Cathey SS, Leroy JG, Wood T, Eaves K, Simensen RJ, Kudo M (2010). Phenotype and genotype in mucolipidoses II and III alpha/beta: a study of 61 probands. J Med Genet.

[20] HGMD® Professional 2016.1. http://portal.biobase-international.com/hgmd/pro/search_gene.php. Accessed 1 Sept 2016.

[21] Plante M, Claveau S, Lepage P, Lavoie EM, Brunet S, Roquis D (2008). Mucolipidosis II: a single causal mutation in the N-acetylglucosamine-1-phosphotransferase gene (GNPTAB) in a French Canadian founder population. Clin Genet.

[22] Lund TC, Cathey SS, Miller WP, Eapen M, Andreansky M, Dvorak CC (2014). Outcomes after hematopoietic stem cell transplantation for children with I-cell disease. Biol Blood Marrow Transplant.

[23] Zhan T, Cui X, Xing X, Ren A, Gan G, Liu Y (2011). Mucolipidosis in a Chinese family with compound heterozygous mutations at the GNPTAB gene. Clin Chim Acta.

[24] Ma GC, Ke YY, Chang SP, Lee DJ, Chen M (2011). A compound heterozygous GNPTAB mutation causes mucolipidosis II with marked hair color change in a Han Chinese baby. Am J Med Genet A.

